# Domain generalisation challenges in breast cancer molecular classification using foundation models: a cross-cohort exploratory study

**DOI:** 10.1007/s11517-026-03590-4

**Published:** 2026-05-11

**Authors:** Jesus Fernandez-Romero, Pablo Ramos-Berciano, Manuel Perez-Perez, David Benavides, Antonio Robles-Frias, Jorge Garcia-Gutierrez, Laura Macias-Garcia

**Affiliations:** 1 Department of Computer Languages and Systems, ETSII, University of Seville, Av. Reina Mercedes s/n, 41012 Seville, Andalusia Spain; 2 UGC Pathology, Hospital Universitario Virgen de Valme, Ctra. de Cádiz Km. 548, 41004 Seville, Andalusia Spain; 3 Department of Normal and Pathological Cytology and Histology, Faculty of Medicine, University of Seville, Av. Doctor Fadriani s/n, 41009 Seville, Andalusia Spain

**Keywords:** Breast cancer, Computational pathology, Domain shift, External validation, Foundation models

## Abstract

**Abstract:**

Molecular classification guides breast cancer treatment, but PAM50 and immunohistochemistry (IHC) remain costly and unavailable in many settings. Foundation models (FMs) combined with multiple instance learning (MIL) show promise for predicting molecular subtypes from haematoxylin-and-eosin-stained slides, yet most studies report only internal validation. This study evaluates FMs with MIL across cohorts and identifies factors associated with domain-induced performance degradation. We evaluate 13 FMs and 3 complementary MIL architectures for PAM50 subtyping and IHC biomarker prediction using cross-validation on TCGA-BRCA ($\boldsymbol{n=1,079}$) and external validation on CPTAC-BRCA ($\boldsymbol{n=120}$). Virchow v2 achieves the best overall performance but exhibits severe degradation upon external validation, consistent across all three MIL architectures especially for HER2-enriched and Normal-like PAM50 subtypes and HER2-positive IHC prediction. Four hypothesised domain shift factors are quantified through exploratory regression analysis to explain relative performance drop (RPD). Staining variability, feature space divergence and morphological separability reach significance in univariate analysis, whilst prevalence shift does not. Staining variability and feature space divergence as covariate-level factors jointly account for 80.0% of RPD variance in the most parsimonious multivariate model ($\boldsymbol{R^2=0.800}$, $\boldsymbol{R^2_{\text {adj}}=0.750}$). Although based on a limited number of class-level observations and therefore exploratory in nature, these findings highlight the need for domain generalisation strategies targeting covariate shift, even when specialised FMs are used as feature encoders.

**Graphical abstract:**

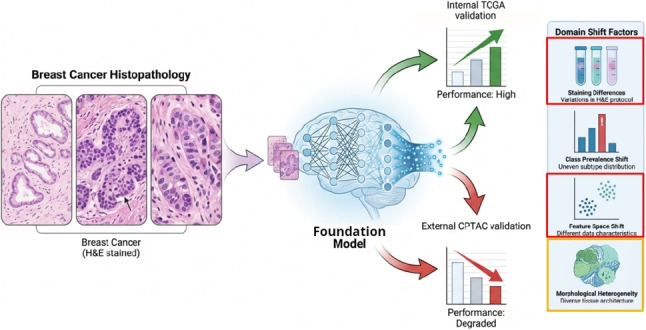

**Supplementary Information:**

The online version contains supplementary material available at 10.1007/s11517-026-03590-4.

## Introduction

Breast cancer (BC) remains one of the leading causes of mortality among women worldwide [1]. Molecular classification plays a crucial role in treatment selection and prognosis, with the PAM50 gene expression assay representing the gold standard for identifying intrinsic molecular subtypes (Luminal A, Luminal B, HER2-enriched, Basal-like, and Normal-like) [2]. Each subtype exhibits distinct biological behaviour and therapeutic responses [3]. However, the high cost and limited availability of molecular testing mean that immunohistochemistry (IHC) remains the primary method for classification in clinical practice [4, 5].

Recent advances in deep learning (DL) have prompted research into predicting molecular subtypes directly from haematoxylin and eosin (H&E)-stained whole slide images (WSIs) to reduce time and economic costs. Multiple instance learning (MIL)—a weakly supervised paradigm combining patch-level feature extraction with slide-level aggregation—has emerged as a leading approach for WSI classification [6]. The development of foundation models (FMs), large-scale models pre-trained on extensive pathology image repositories, has further enhanced MIL performance by providing robust visual representations [7]. FMs and MIL approaches have already shown potential in BC-related tasks such as HER2 score prediction [8] and molecular subtyping classification [7].

Beyond performance metrics, interpretability is essential for clinical adoption of DL systems [9, 10]. Attention-based MIL models such as Clustering-constrained Attention MIL (CLAM) [11] offer inherent interpretability by identifying image regions that contribute most to predictions. However, limited research has systematically evaluated whether these models learn biologically meaningful histomorphological patterns that remain consistent across independent cohorts, or whether apparent success reflects overfitting to institution-specific characteristics. Indeed, most published studies [4, 12] evaluate performance exclusively on internal validation sets from the same institution used for training, without testing on independent external cohorts, failing to assess domain generalisation—the ability to maintain performance when deployed on data from different institutions or technical protocols. Domain shift—systematic differences in data characteristics between training and deployment environments—is well-documented in medical imaging [13], encompassing technical factors (tissue preparation, scanning equipment, staining procedures) [14] and population factors (patient demographics, disease prevalence) [15]. Recent work has explored FMs for BC molecular classification [7] and demonstrated the importance of cross-domain generalisation [16], but systematic external validation with explicit characterisation of domain shift mechanisms remains absent.

To our knowledge, this is the first study to systematically characterise the sources of domain-induced performance degradation in the context of breast cancer molecular subtype classification from H&E-stained slides. To this end, this study evaluates FM-based MIL molecular classification with rigorous external validation and provides an exploratory characterisation of domain shift factors. Our contributions include: (1) A systematic evaluation of 13 FMs (12 state-of-the-art plus ResNet-50 baseline) on PAM50 subtyping and IHC prediction using patient-stratified validation (TCGA-BRCA) and independent external validation (CPTAC-BRCA) with class-imbalance-aware metrics (macro-F1, PR-AUC). (2) An evaluation of the best FM in the previous step using three optimised complementary MIL strategies. (3) A quantification and statistical exploratory analysis of four hypothesised factors that may affect cross-cohort generalisation performance: (i) staining variability, (ii) class prevalence shifts, (iii) feature space divergence, and (iv) lack of morphological separability.

## Methods

### Datasets

We used WSIs from two public breast cancer datasets. The Cancer Genome Atlas Breast Invasive Carcinoma (TCGA-BRCA, hereafter TCGA) [17] served as the internal cohort, whilst the Clinical Proteomic Tumor Analysis Consortium Breast Cancer (CPTAC-BRCA, hereafter CPTAC) [18] served as the external validation cohort.

From TCGA, we selected cases based on supplementary annotations from Thennavan et al. [19], which included PAM50 molecular subtypes and IHC biomarker status (ER, PR, HER2). To ensure consistency with CPTAC (which comprises flash-frozen samples), we excluded formalin-fixed paraffin-embedded specimens. This yielded 1522 slides from 1079 patients with PAM50 annotations. IHC biomarker status was available for 1455 (ER), 1452 (PR) and 1482 (HER2) slides.

From CPTAC, we selected 387 flash-frozen slides from 120 patients with complete PAM50 and IHC annotations as reported in Krug et al. [20]. Specifically, 379, 367 and 387 slides had complete annotations for ER, PR and HER2 status, respectively.

### Experimental pipeline

Figure S1 depicts the experimental pipeline: (a) patch extraction, (b) Monte Carlo cross-validation (MCCV) with baseline CLAM on TCGA, (c) external validation on CPTAC with baseline MIL, and (d) domain shift analysis based on the results of the combination of the best FM and three optimised MIL in PathBench-MIL [21]. To ensure reproducibility, all scripts are publicly available and based on a fork of the CLAM repository [22] and the original PathBench-MIL [23].

#### Foundation model evaluation

WSIs were preprocessed using CLAM [22] as baseline MIL which included its own tissue segmentation. Valid 512$\times$512 pixel patches were extracted at maximum magnification (40$\times$) using a sliding window approach for visual inspection. Later, the patches were approximately adapted to the most common 256$\times$256 at 20$\times$ format by the FMs which internally rescale patches to their native resolution (e.g., Virchow v2: 224$\times$224). We compared 13 feature extractors: 12 state-of-the-art FMs (July 2023–January 2025) plus ResNet-50 ImageNet baseline (see Table S1). All models were frozen feature extractors for CLAM. Hyperparameters were determined by grid search and the final selection is detailed in Table S2.

We performed patient-stratified MCCV with 10 random splits on TCGA as internal validation. In each iteration, patients were randomly partitioned into 80% training, 10% validation (for early stopping) and 10% test. Patient-level stratification ensured that all slides from the same patient remained in the same fold, preventing data leakage. To address class imbalance during training, we used class-weighted cross-entropy loss with weights inversely proportional to class frequencies. Performance was evaluated using class-imbalance-aware metrics computed with scikit-learn [24]: macro F1-score for PAM50 and PR-AUC for receptor status prediction (ER, PR, HER2).

To evaluate domain generalisation, each FM-CLAM model was trained on the entire TCGA cohort (85% training, 15% validation) and tested on CPTAC. Models were trained using the same hyperparameters as internal validation (Table S2), with early stopping based on validation loss. The best-performing checkpoint was used for testing on CPTAC.

Based on superior internal and external validation performance across all tasks, we selected the FM with the best mean ranking across MCCV and HO for subsequent domain shift characterisation study.

#### Multiple instance learning comparison

To assess the sensitivity of domain shift findings to MIL aggregator architecture, we evaluated three complementary aggregation strategies representing distinct architectural families: CLAM [11] (attention-based) with multiple attention branches, TransMIL [25] (transformer-based), and DSMIL [26] (dual-stream). This selection was motivated by the need to cover architecturally diverse aggregation paradigms whilst remaining computationally feasible. Models were trained using 10-fold cross-validation (CV) on TCGA and evaluated on CPTAC as an independent hold-out cohort. Hyperparameters for each architecture were independently optimised (see Table S3) on the training set using the PathBench-MIL framework [21] with Optuna [27], employing a pruning strategy over 50 trials, maximising mean average precision on the validation set (10% of training). Specifically, embedding dimension ($z_{\text {dim}} \in [32, 512]$) and bag size ([8, 256]) were optimised whilst all other settings were held constant across architectures following the CLAM baseline parametrisation: cross-entropy loss, ReLU activation, and Adam optimiser. Tile size was fixed at $256\times 256$ px / 128 $\mu$m ($\approx$20$\times$) as this resolution is optimal for most FMs. No stain normalisation was initially applied.

#### Performance degradation quantification

To enable comparison between F1-score (PAM50) and PR-AUC (IHC) across different tasks, we computed Relative Performance Degradation (RPD) as the proportional difference between internal validation (CV) and external validation (HO) performance. For each of the five PAM50 molecular subtypes (Luminal A, Luminal B, HER2-enriched, Basal-like, Normal-like) and IHC biomarker status classes (ER+, ER-, PR+, PR-, HER2+, HER2-), RPD was computed as:

1 $$\begin{aligned} \text {RPD}_{Q,c} = {\left\{ \begin{array}{ll} \dfrac{Q_c^{\text {CV}} - Q_c^{\text {HO}}}{Q_c^{\text {CV}}}, & \text {if } Q_c^{\text {CV}} > 0,\\ n.d., & \text {otherwise.} \end{array}\right. } \end{aligned}$$

where, for label *c* and a performance metric *Q* (i.e., F1-score or PR-AUC), $Q_c^{\text {CV}}$ and $Q_c^{\text {HO}}$ represent the mean performance metric for label *c* across internal (10-fold CV) and external validation (HO), respectively. For example, if a model achieves a class-level F1-score of 0.60 on internal validation and 0.42 on external validation, the RPD is $(0.60 - 0.42) / 0.60 = 0.30$, indicating a 30% relative drop in performance upon external deployment. An RPD of 0 indicates no degradation, an RPD of 1.0 indicates complete performance collapse, and negative values indicate improved performance on the external cohort. For the domain shift regression analysis, RPD values for every label were computed as the mean across the three optimised MIL architectures, yielding more robust per-class estimates of cross-cohort performance degradation.

#### Stain normalisation robustness

To assess whether staining variability contributed to domain shift, we applied Macenko normalisation [28] to all patches during pre-processing prior to feature extraction. We used the PathBench-MIL framework, which leverages the SlideFlow [29] stain normalisation pipeline with its population-level preset (v3). This preset defines the stain matrix target — a $3 \times 2$ matrix encoding the H and E staining vectors in RGB space — and the reference maximum concentrations ([1.766, 1.280]), estimated as the average of Macenko decomposition parameters across approximately 50,000 patches drawn from 450 TCGA slides. Normalisation was applied patch by patch during feature bag generation, immediately prior to feature extraction with Virchow v2, transforming each patch so that its H&E staining characteristics matched the TCGA population reference.

We trained the three optimised MIL architectures on normalised TCGA using identical hyperparameters to those used in Section 2.2.3 and evaluated them on normalised CPTAC. For each molecular class *c*, we quantified the normalised performance difference per class ($\Delta n_c$) as:

2 $$\begin{aligned} \Delta n_c= {Perf}_c^{normalised} - {Perf}_c^{original} \end{aligned}$$

where $\text {Perf}_c^{\text {normalised}}$ and $\text {Perf}_c^{\text {original}}$ represent per-class performance (F1-score for PAM50, PR-AUC for IHC) on HO, averaged across the three optimised MIL architectures, for models trained on normalised and original images, respectively.

#### Distributional shift analysis

We quantified distributional shift and its relationship to generalisation failure. For each class in a classification task, we computed class prevalence in both cohorts:

3 $$\begin{aligned} p_c^{\text {TCGA}} = \frac{N_c^{\text {TCGA}}}{N^{\text {TCGA}}}, \quad p_c^{\text {CPTAC}} = \frac{N_c^{\text {CPTAC}}}{N^{\text {CPTAC}}} \end{aligned}$$

where $N^D_c$ is the number of samples of class *c* in a dataset *D* and $N^D$ is the total sample count in *D*. Prevalence shift for each class was computed as:

4 $$\begin{aligned} \Delta p_c = p_c^{\text {CPTAC}} - p_c^{\text {TCGA}} \end{aligned}$$

#### Feature space consistency analysis

We evaluated the consistency of the best FM embeddings across cohorts using the most diagnostically relevant patches identified by each MIL architecture. For each WSI in both TCGA and CPTAC, we used the attention weights produced during the HO evaluation of each of the three optimised MIL models to select the top $K=8$ patches per WSI. For CLAM, the attention column corresponding to the ground-truth class label was used; for DSMIL and TransMIL, the single attention vector was used.

Within each molecular class, patches were grouped by ground-truth label (PAM50 subtype or IHC biomarker status) and dataset. Embeddings were loaded for all selected patches and class centroids were computed as the arithmetic mean:

5 $$\begin{aligned} \boldsymbol{\mu }_c^{D} = \frac{1}{M} \sum _{i=1}^{M} \textbf{z}_i \end{aligned}$$

where $D \in \{\text {TCGA}, \text {CPTAC}\}$, *M* is the total number of selected patches from WSIs labelled as class *c* in dataset *D* according to ground-truth annotations, and $\textbf{z}_i$ is the patch-level embedding. The cosine distance between corresponding centroids was then computed as:

6 $$\begin{aligned} d_c = 1 - \frac{\boldsymbol{\mu }_c^{\textrm{TCGA}} \cdot \boldsymbol{\mu }_c^{\textrm{CPTAC}}}{\Vert \boldsymbol{\mu }_c^{\textrm{TCGA}}\Vert \, \Vert \boldsymbol{\mu }_c^{\textrm{CPTAC}}\Vert } \end{aligned}$$

For the domain shift regression analysis, $d_c$ was computed independently for each of the three optimised MIL architectures and the mean value was used as the per-class estimate of feature space divergence.

#### Cross-cohort morphological analysis

We analysed the histomorphological characteristics of patches selected by the baseline CLAM model for each molecular classification task (PAM50 subtypes and IHC biomarker status) and dataset. The baseline CLAM model was used for two reasons: first, it was employed in the FM selection phase and therefore identified the best-performing feature extractor; second, patches were extracted at 512$\times$512 pixels at 40x magnification — double the 20x standard resolution — which provides substantially greater morphological detail and facilitates pathologist assessment of histological features. For each class, we followed the steps in Section 2.2.6 and selected the 25 patches closest to the class centroid in the best FM embedding space, identifying the most representative patches for morphological annotation. This yielded 125 PAM50 patches (5 subtypes $\times$ 25 patches) and 150 IHC patches (3 biomarkers $\times$ 2 statuses $\times$ 25 patches) per cohort.

Two board-certified pathologists with more than five years of experience in breast cancer diagnostics, blinded to ground-truth molecular labels and model predictions, independently annotated the selected patches. For every patch, the following histomorphological features were recorded: Tubule Formation (ordinal: 1–3), Nuclear Pleomorphism (ordinal: 1–3), Mitotic Activity (number of mitoses detected), Tumour Necrosis (binary: present/absent), Lymphocytic Infiltrate (binary: present/absent), and Polymorphonuclear (PMN) Infiltrate (binary: present/absent). Given the inherent subjectivity of morphological scoring [30], no formal consensus protocol was applied; instead, the definitive score for each patch and feature was taken as the arithmetic mean of both pathologists’ independent assessments, a standard approach for reducing random annotation error whilst preserving the original sample size and observation independence. Inter-rater reliability was assessed between the two pathologists on the same set of 275 representative patches, aligned by image identifier, according to Landis & Koch [31]. Weighted $\kappa _w$ (linear weights) was computed for ordinal features (Tubule Formation, Nuclear Pleomorphism, Mitotic Activity) and Cohen’s $\kappa$ for binary features (Necrosis, Lymphocytic Infiltrate, PMN).

We statistically analysed the histomorphological characteristics to quantify biological domain shift. We compared histomorphological feature distributions between TCGA and CPTAC patches using Mann-Whitney U tests, with corresponding effect sizes (rank-biserial correlation $r_{rb}$). Multiple comparisons were corrected using the Benjamini–Hochberg (BH) procedure at $\alpha = 0.05$ within each class pair. Analyses were performed separately for each pair of molecular classes to construct morphological similarity matrices: a 5$\times$5 matrix for PAM50 subtypes, and three 2$\times$2 matrices for ER, PR and HER2 status, respectively. Each cell $(c,c')$ in a similarity matrix quantified the sum of effect sizes for statistically significant features only (BH-adjusted $q < 0.05$) between class *c* and class $c'$. For each class pair, we computed the sum of absolute effect sizes across all statistically significant histomorphological features as:

7 $$\begin{aligned} B_{c,c'} = \sum _{i=1}^{6}|r_{rb,i}^{(c,c')}| \end{aligned}$$

where $B_{c,c'}$ quantifies absolute rank-biserial correlations for the features, computed when comparing class *c* against class $c'$. The diagonal elements $B_{c,c}$ (or simply $B_c$) represent within-class morphological heterogeneity between cohorts (how different class *c* appears when comparing TCGA vs. CPTAC), whilst off-diagonal elements $B_{c,c'}$ ($c' \ne c$) capture cross-class morphological similarity (how similar class $c'$ in CPTAC is to other class *c* in TCGA).

To assess whether morphological separability predicted generalisation performance, we computed a morphological separability metric for each class:

8 $$\begin{aligned} \tilde{B}_c = \min _{d \ne c} B_{c,d} - B_c \end{aligned}$$

This metric quantifies the difference between the minimum cross-class morphological similarity (smallest off-diagonal $B_{c,d}$) and within-class morphological consistency (diagonal $B_c$). Positive values indicate that class *c* is morphologically more stable within itself across cohorts than it is similar to any other class, suggesting preserved discriminative boundaries that may facilitate generalisation. Negative values indicate that class *c* in CPTAC is morphologically more similar to at least one other class in TCGA than to itself, suggesting morphological confusion that may impair cross-cohort classification.

#### Statistical analysis of domain shift factors

We performed an exploratory linear regression analysis integrating findings from all four domain shift characterisation analyses across both PAM50 molecular subtyping and IHC biomarker prediction ($n=11$ classes total).

The regression model pooled observations across both tasks (PAM50 and IHC). This pooling was justified on three grounds. First, the dependent variable RPD was a normalised, dimensionless measure of proportional performance drop, computed separately for each class using its corresponding metric (macro F1-score for PAM50, PR-AUC for IHC), which ensured comparability across tasks independently of the underlying metric scale. Second, the domain shift factors under investigation were defined at the class level and were hypothesised to operate through mechanisms independent of the specific biological task. Third, pooling across tasks increased the number of observations from 5 (PAM50 subtypes only) to 11, providing greater statistical power for the exploratory analysis.

To control for multiple comparisons, the Benjamini–Hochberg (BH) procedure was applied separately within each section of the analysis (univariate Spearman tests, univariate OLS slope tests, multivariate model tests, and collinearity tests). The BH procedure ranked the *m* *p*-values within each family and rejected hypothesis $H_{(i)}$ if $p_{(i)} \le (i/m)\,\alpha$, controlling the false discovery rate at level $\alpha = 0.05$. The resulting adjusted *p*-value was denoted *q*. A factor was considered statistically significant for inclusion in the multivariate model if it reached $q < 0.05$ under BH correction in the univariate analysis.

Univariate associations between each domain shift factor and RPD were assessed via simple OLS regression, reporting Pearson *r* (linear association), coefficient of determination $R^2$, regression slope $\beta$, and its *q*-value. We also reported Spearman $\rho$ with its corresponding *q*-value (monotonic association, more robust given $n=11$ and potential non-normality of factors).

Factors reaching significance in univariate analysis were considered as candidates for the multivariate model. A multivariate OLS model was then fitted using the most parsimonious combination of significant factors. Pairwise collinearity between the candidate factors (see Figure was assessed via Pearson *r*, Spearman $\rho$ and the variance inflation factor (VIF), computed as $1/(1-R^2)$ from a bivariate regression. Model fit was characterised by $R^2$, adjusted $R^2$, *F*-statistic and its *q*-value. Individual coefficients were reported with their standard error, *t*-statistic and *q*-value.

The general formulation of the regression model with RPD as the dependent variable and the four domain shift factors as independent variables was (though only those reaching univariate significance were considered for the final multivariate model):

9 $$\begin{aligned} \text {RPD}_c = \beta _0 + \beta _1 \Delta n_c + \beta _2 \Delta p_c + \beta _3 d_c + \beta _4 \tilde{B}_c + \varepsilon \end{aligned}$$

The coefficient of determination ($R^2$) quantified the proportion of variance in performance degradation explained by each model.

## Results

Table 1 presents performance metrics and rankings (1=best, 13=worst) for each FM model when the baseline CLAM was applied. Virchow v2 achieved the best mean ranking (2.00), followed by Prov-GigaPath (4.13), H-optimus-0 (4.25), and UNI-2 (4.25). Earlier models (CTransPath: 7.00, RetCCL: 9.63) and the ResNet-50 baseline (12.75) ranked substantially lower, confirming Virchow v2 selection for the domain shift characterisation.

**Table 1:** Performance metrics with relative rankings. For PAM50, macro F1-scores are reported; for ER, PR and HER2, PR-AUC values are shown. First row per model shows mean values; second row shows standard deviation (MCCV only) and rankings in parentheses (1=best, 13=worst). Mean Rank shows average ranking across all eight evaluations

	PAM50		ER		PR		HER2		Mean
Model	MCCV	HO	MCCV	HO	MCCV	HO	MCCV	HO	Rank
ResNet-50	0.342	0.218	0.933	0.722	0.822	0.595	0.326	0.104	12.75
	±0.030 (13)	(13)	±0.030 (13)	(13)	±0.045 (13)	(13)	±0.102 (11)	(13)	
CTransPath	0.446	0.342	0.962	0.870	0.845	0.757	0.395	0.156	7.00
	±0.056 (10)	(5)	±0.021 (8)	(8)	±0.040 (8)	(8)	±0.127 (3)	(6)	
RetCCL	0.414	0.272	0.956	0.804	0.837	0.736	0.368	0.130	9.63
	±0.050 (11)	(12)	±0.026 (10)	(10)	±0.042 (9)	(9)	±0.095 (6)	(10)	
CONCH	0.493	0.335	0.957	0.885	0.853	0.777	0.306	0.190	7.13
	±0.052 (7)	(6)	±0.015 (9)	(6)	±0.029 (7)	(7)	±0.086 (12)	(3)	
UNI	0.527	0.365	0.967	0.885	0.870	0.833	0.396	0.148	4.38
	±0.044 (4)	(2)	±0.025 (5)	(7)	±0.029 (4)	(3)	±0.105 (2)	(8)	
Prov-GigaPath	0.504	0.379	0.967	0.900	0.875	0.822	0.368	0.160	4.13
	±0.050 (6)	(1)	±0.018 (6)	(4)	±0.029 (2)	(4)	±0.089 (5)	(5)	
Hibou-B	0.457	0.289	0.964	0.803	0.835	0.696	0.354	0.133	9.63
	±0.059 (8)	(11)	±0.025 (7)	(11)	±0.043 (10)	(12)	±0.109 (9)	(9)	
Hibou-L	0.399	0.297	0.952	0.858	0.826	0.697	0.246	0.107	11.38
	±0.034 (12)	(10)	±0.022 (12)	(9)	±0.040 (12)	(11)	±0.042 (13)	(12)	
H-optimus-0	0.565	0.304	0.973	0.897	0.883	0.803	0.377	0.153	4.25
	±0.053 (2)	(9)	±0.015 (1)	(5)	±0.039 (1)	(5)	±0.096 (4)	(7)	
Virchow v2	0.542	0.358	0.972	0.916	0.874	0.862	0.399	0.219	2.00
	±0.041 (3)	(3)	±0.014 (2)	(2)	±0.035 (3)	(1)	±0.115 (1)	(1)	
Phikon v2	0.508	0.345	0.971	0.906	0.861	0.802	0.359	0.191	4.63
	±0.035 (5)	(4)	±0.017 (3)	(3)	±0.036 (6)	(6)	±0.106 (8)	(2)	
Musk	0.450	0.305	0.955	0.774	0.832	0.700	0.364	0.126	9.88
	±0.074 (9)	(8)	±0.018 (11)	(12)	±0.038 (11)	(10)	±0.106 (7)	(11)	
UNI-2	0.575	0.325	0.969	0.917	0.868	0.858	0.353	0.164	4.25
	±0.061 (1)	(7)	±0.019 (4)	(1)	±0.029 (5)	(2)	±0.098 (10)	(4)	

**Fig. 1:**
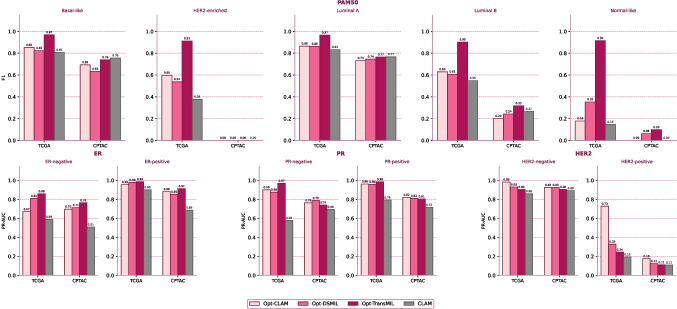
PathBench-MIL results on validation and testing for Optuna-optimised CLAM, DSMIL and TransMIL, compared with baseline CLAM used in Table 1

Figure 1 shows per-class performance for the three Optuna-optimised MIL architectures and the non-optimised baseline CLAM on both TCGA and CPTAC. Detailed per-class performance values and RPD calculations for the three optimised architectures are provided in Table S4. The three optimised architectures consistently outperformed the baseline across most classes. Nevertheless, degradation patterns on CPTAC were remarkably consistent across all architectures: HER2-enriched exhibited complete performance collapse (RPD = 1.000 across all models); Normal-like showed severe degradation across all models despite TransMIL achieving the highest internal performance on TCGA; and Luminal B showed high RPD across all architectures. Luminal A and Basal-like maintained relatively stable performance. For IHC tasks, ER and PR showed moderate degradation, whilst HER2-positive exhibited substantial performance drop.

Table 2 summarises the four domain shift factors and RPD for each of the 11 classes. Stain normalisation showed a heterogeneous effect across classes and architectures (Table S5), with HER2-enriched showing no change under any architecture ($\Delta n_c = 0.000$). Class prevalence differed between cohorts across all tasks (Table S6), with the largest shifts observed for ER status and Basal-like, and the smallest for Normal-like. Cosine centroid distances (Table S7) were highest for HER2-enriched ($d_c = 0.197$) and lowest for ER-positive ($d_c = 0.105$), with DSMIL consistently yielding higher distances than CLAM and TransMIL. Morphological separability values ($\tilde{B}_c$) ranged from $-1.232$ (Normal-like) to $+2.642$ (ER-positive), derived from the cross-cohort morphological analysis (Table S8), in which inter-rater agreement between the two pathologists ranged from slight to fair ($\kappa _w = 0.152$–0.289 for ordinal features; $\kappa = 0.177$–0.321 for binary features; Table S9).

**Table 2:** Domain shift factors ($\Delta n$, *d*, $\Delta p$ and $\tilde{B}$) as independent variables for statistical analysis with relative performance degradation (RPD) as dependent variable. $\Delta n_c$, $d_c$ and $RPD_c$ for every class *c* are averaged across the three Optuna-optimised MIL models (CLAM, DSMIL and TransMIL)

Task	Class	$\Delta n_c$	$\Delta p_c$	$d_c$	$\tilde{B}_c$	RPD
PAM50	Basal-like	$+0.015$	$+0.132$	0.139	$-0.125$	$+0.219$
	HER2-enriched	$+0.000$	$+0.025$	0.197	$-0.904$	$+1.000$
	Luminal A	$-0.020$	$-0.075$	0.123	$+0.574$	$+0.166$
	Luminal B	$+0.067$	$-0.066$	0.149	$+0.693$	$+0.644$
	Normal-like	$+0.061$	$-0.016$	0.147	$-1.232$	$+0.906$
ER	ER-negative	$-0.053$	$+0.220$	0.136	$+1.445$	$+0.063$
	ER-positive	$-0.038$	$-0.220$	0.105	$+2.642$	$+0.093$
PR	PR-negative	$-0.028$	$+0.143$	0.112	$+1.080$	$+0.163$
	PR-positive	$-0.032$	$-0.143$	0.118	$+1.558$	$+0.161$
HER2	HER2-negative	$+0.006$	$+0.099$	0.106	$-0.296$	$+0.021$
	HER2-positive	$+0.035$	$-0.099$	0.115	$+0.419$	$+0.643$

Table 3 presents the results of the univariate and multivariate regression analyses. In univariate analysis (Fig. S2), $\Delta n$, *d* and $\tilde{B}$ reached BH-corrected significance, whilst $\Delta p$ did not ($q=0.615$). The most parsimonious multivariate model, RPD $\sim \Delta n + d$, achieved $R^2=0.800$ ($R^2_{\text {adj}}=0.750$, $q=0.005$), with both factors retaining significant associations with RPD under BH correction. Once $\Delta n$ and *d* were included, $\tilde{B}$ contributed no independent predictive power ($q=0.881$, $\Delta R^2<0.001$) and was excluded from the final model. Collinearity analysis (Fig. S3) showed moderate correlation between $\Delta n$ and $\tilde{B}$ ($\rho =-0.691$, $q=0.056$) and no significant collinearity between $\Delta n$ and *d* ($\rho =+0.455$, $q=0.160$).

**Table 3:** Statistical models predicting RPD from domain shift factors ($n=11$ molecular classes). BH correction applied separately within each section. $^{*}q<0.05$, $^{\dagger }q<0.10$

Univariate models						
Model	*r*	$\rho$	$q(\rho )$	$R^2$	$\beta$	*q*-value
*d*	$+0.759$	$+0.755$	0.029$^{*}$	0.577	$+10.110$	0.027$^{*}$
$\Delta n$	$+0.692$	$+0.673$	0.047$^{*}$	0.479	$+6.160$	0.037$^{*}$
$\tilde{B}$	$-0.651$	$-0.573$	0.087$^{\dagger }$	0.424	$-0.204$	0.040$^{*}$
$\Delta p$	$-0.171$	$-0.109$	0.750	0.029	$-0.449$	0.615
Multivariate model						
Model	$R^2$	Adj. $R^2$	*F*-stat	*q*-value		
RPD $\sim \Delta n + d$	0.800	0.750	16.03	0.005$^{*}$		
	$\beta$	Std Error	*t*-value	*q*-value	$\Delta R^2$	
*d*	$+7.9728$	2.2215	$+3.589$	0.011$^{*}$	0.322	
$\Delta n$	$+4.4456$	1.4857	$+2.992$	0.017$^{*}$	0.224	
$\tilde{B}$	$+0.0125$	0.0805	$+0.156$	0.881	0.001	
Factor collinearity						
Pair	Pearson *r*	Spearman $\rho$	$q(\rho )$	VIF		
$d \sim \tilde{B}$	$-0.583$	$-0.482$	0.160	1.515		
$\Delta n \sim d$	$+0.321$	$+0.455$	0.160	1.115		
$\Delta n \sim \tilde{B}$	$-0.639$	$-0.691$	0.056$^{\dagger }$	1.691		

## Discussion

Our systematic evaluation of 13 FMs with the baseline CLAM revealed that Virchow v2 achieved the best overall performance (Table 1), a finding independently corroborated by Ma et al. [7] for BC molecular classification using a generic ABMIL framework. However, this ranking masked severe degradation patterns upon external validation, including complete performance collapse for HER2-enriched and Normal-like PAM50 subtypes, and substantial degradation in HER2-positive IHC prediction. These results contrast with recent FM studies reporting successful BC molecular classification, which either excluded difficult-to-classify subtypes [4], evaluated on overlapping institutional datasets [32], or reported only imbalance-unaware performance metrics without per-class external validation [33].

These degradation patterns were not specific to the baseline CLAM architecture. Their consistency across three complementary Optuna-optimised MIL architectures (Table S4) suggests that the observed limitations were rooted in the feature representations produced by the FM and in the nature of the classification task, rather than in any specific MIL design choice. Critically, optimisation on the internal validation set did not translate into improved cross-cohort generalisation in most cases, suggesting that the performance gap between internal and external evaluation reflects a genuine domain generalisation problem rather than a suboptimal training configuration.

The domain shift metrics computed in this study showed differential associations with cross-cohort performance degradation, collectively pointing to covariate-level factors as the primary drivers in this setting. Feature space divergence (*d*) showed the strongest univariate association ($R^2=0.577$), suggesting that even modest increases in centroid distance between cohorts were associated with disproportionately large performance drops, consistent with the use of feature distribution comparisons as indicators of covariate shift severity [34]. Staining variability benefit ($\Delta n$) showed a positive linear association with RPD, consistent with the well-documented role of staining variation as a source of domain shift in computational pathology [34, 35]; the heterogeneous and class-dependent effect of Macenko normalisation suggests that Virchow v2 did not fully disentangle biological morphology from colour-dependent features for all molecular classes [34]. In contrast, prevalence shift ($\Delta p$) showed no significant association with RPD; whilst prevalence shift (also termed prior shift in the domain generalisation literature) has been proposed as a source of domain-induced degradation [15], the literature identifies covariate shift as the primary barrier to cross-site generalisation [34], a pattern consistent with our findings. Finally, morphological separability ($\tilde{B}$) showed a negative association with RPD, consistent with the definition of class-conditional shift [34], whereby classes whose morphological characteristics diverge across cohorts (i.e., lower separability in the external cohort) occupy less discriminative regions of the embedding space, rendering their classification boundaries more susceptible to domain-induced degradation.

In the multivariate model, $\Delta n$ and *d* jointly explained 80% of the variance in RPD ($R^2=0.800$, $R^2_{\text {adj}}=0.750$), with *d* contributing the larger unique share ($\Delta R^2=0.322$ vs. 0.224 for $\Delta n$). Despite its univariate significance, $\tilde{B}$ contributed no independent predictive power once $\Delta n$ and *d* were included ($\Delta R^2<0.001$). Collinearity analysis revealed a moderate negative association between $\tilde{B}$ and $\Delta n$ ($\rho =-0.691$), suggesting that classes with lower morphological separability tend to show greater staining variability benefit — that is, their cross-cohort degradation may be partly mediated through colour-dependent rather than purely structural features. A weaker negative association was also observed between $\tilde{B}$ and *d* ($\rho =-0.482$), suggesting that morphologically less separable classes also tend to occupy more divergent positions in the Virchow v2 embedding space across cohorts. Together, these collinearity patterns suggest that $\tilde{B}$, whilst associated with RPD in univariate analysis, may capture aspects of domain shift that are already reflected in $\Delta n$ and *d*, rendering it redundant in the joint model.

To our knowledge, this is the first study to systematically characterise the limitations of FM-based molecular subtype classification using MIL from H&E under cross-cohort evaluation. The identification of covariate-level factors — staining variability and feature space divergence — as the primary drivers of performance degradation opens a research direction for domain adaptation techniques operating at the feature representation level, such as Low-Rank Adaptation [36], Specialised Model-Sample Matching [37], or Mix-of-Adapters [38], as promising strategies to improve cross-cohort generalisation.

### Limitations of this study

This study provides an exploratory characterisation of domain-induced performance degradation in BC molecular classification but several limitations should be acknowledged:

- TCGA and CPTAC datasets were restricted to flash-frozen tissue, limiting generalisability to formalin-fixed paraffin-embedded (FFPE) samples, which are more common in clinical workflows. This assumption implies that the covariate-level factors identified may not fully capture the sources of domain shift in FFPE-based cohorts.
- The benchmark did not incorporate FM releases from early 2025 (e.g., H-optimus-1), whose weights were unavailable at the time of experimentation; conclusions may evolve as newer FMs are evaluated. Furthermore, FM selection was performed using the baseline CLAM architecture, and it is possible that a different FM ranking would emerge under optimised MIL configurations, although the independent corroboration of Virchow v2 superiority by Ma et al. [7] partially mitigates this concern.
- The regression analysis was limited by sample size ($n=11$ classes) and should be interpreted as exploratory. PAM50 and IHC were pooled without task type as a covariate; this assumption implies that the identified factors operate through class-level mechanisms independent of the biological task, which remains unverified. Findings should be interpreted as hypothesis-generating.
- Inter-rater agreement between pathologists was modest and the limited patch sample ($n=25$ per class) may have reduced the robustness of $\tilde{B}$. Whilst these agreement levels are lower than those reported in the literature for standard histological grading [30], the annotation task in our study — characterising morphological features of patches selected for molecular subtype representativeness — is considerably more subjective than conventional grading. Nevertheless, this fact represents a source of uncertainty in the morphological separability estimates that should be considered when interpreting the univariate association between $\tilde{B}$ and RPD.

## Conclusions

This study provided the first systematic characterisation of domain-induced performance degradation in FM-based MIL classification of BC molecular subtypes. Virchow v2 achieved the best overall performance across 13 evaluated FMs but exhibited severe cross-cohort degradation consistent across three optimised MIL architectures, suggesting feature representations rather than aggregation design as the primary source of failure. An exploratory regression analysis identified staining variability and feature space divergence as the primary factors associated with relative degradation ($R^2=0.800$), whilst prevalence shift showed no significant association, highlighting covariate shift as the dominant barrier to cross-cohort generalisation.

Future work should expand the analysis to larger and more diverse cohorts, additional molecular classes (e.g., Ki67 prediction), and FFPE-based datasets to increase statistical power and clinical relevance. Furthermore, domain adaptation strategies operating at the feature representation level represent promising directions for improving cross-cohort generalisation of FM-based molecular classifiers.

## Supplementary Information

Below is the link to the electronic supplementary material.

Supplementary file 1 (pdf 1.76 MB)

## References

[1] Zhang Y, Ji Y, Liu S et al (2025) Global burden of female breast cancer: new estimates in 2022, temporal trend and future projections up to 2050 based on the latest release from GLOBOCAN. J Natl Cancer Center 5(3):287–296. 10.1016/j.jncc.2025.02.002 40693239 10.1016/j.jncc.2025.02.002 PMC12276554

[2] Wallden B, Storhoff J, Nielsen T, et al (2015) Development and verification of the PAM50-based Prosigna breast cancer gene signature assay. BMC Med Genomics 8(54) 10.1186/s12920-015-0129-6 PMC4546262 26297356

[3] Ziegengeist JL, Tan AR (2025) A clinical review of subcutaneous trastuzumab and the fixed-dose combination of pertuzumab and trastuzumab for subcutaneous injection in the treatment of HER2-positive breast cancer. Clin Breast Cancer 25(2):124–132. 10.1016/j.clbc.2024.10.005 10.1016/j.clbc.2024.10.005 39567339

[4] Tafavvoghi M, Sildnes A, Rakaee M et al (2025) Deep learning-based classification of breast cancer molecular subtypes from H&E whole-slide images. J Pathology Inf 16:100410. 10.1016/j.jpi.2024.100410 10.1016/j.jpi.2024.100410 PMC11667687 39720418

[5] Niyas S, Bygari R, Naik R et al (2023) Automated molecular subtyping of breast carcinoma using deep learning techniques. IEEE J Trans Eng Health Med 11:161–169. 10.1109/JTEHM.2023.3241613 10.1109/JTEHM.2023.3241613 PMC9924555 36816095

[6] Huang J, Li G, Kan S et al (2025) An efficient framework based on large foundation model for cervical cytopathology whole slide image screening. Biomed Signal Process Control 107:107859. 10.1016/j.bspc.2025.107859

[7] Ma J, Xu Y, Zhou F et al (2025) PathBench: A comprehensive comparison benchmark for pathology foundation models towards precision oncology. arxiv:2505.20202

[8] Valieris R, Martins L, Defelicibus A et al (2024) Weakly-supervised deep learning models enable HER2-low prediction from H&E stained slides. Breast Cancer Res 26:124. 10.1186/s13058-024-01863-0 39160593 10.1186/s13058-024-01863-0 PMC11331614

[9] Antamis T, Drosou A, Vafeiadis T et al (2024) Interpretability of deep neural networks: A review of methods, classification and hardware. Neurocomputing 601:128204. 10.1016/j.neucom.2024.128204

[10] Dolezal JM, Wolk R, Hieromnimon HM, et al (2023) Deep learning generates synthetic cancer histology for explainability and education. NPJ Precis Onc 7(23). 10.1038/s41698-023-00399-4 10.1038/s41698-023-00399-4 PMC10227067 37248379

[11] Lu MY, Williamson DF, Chen T et al (2021) Data-efficient and weakly supervised computational pathology on whole-slide images. Nat Biomed Eng 5(6):555–570. 10.1038/s41551-020-00682-w 33649564 10.1038/s41551-020-00682-w PMC8711640

[12] Shi J, Sun D, Jiang Z et al (2025) Weakly supervised multi-modal contrastive learning framework for predicting the her2 scores in breast cancer. Comput Med Imaging Graph 121:102502. 10.1016/j.compmedimag.2025.102502 39919535 10.1016/j.compmedimag.2025.102502

[13] Pérez-Núñez J, Rodríguez C, Vásquez-Serpa L et al (2024) The challenge of deep learning for the prevention and automatic diagnosis of breast cancer: a systematic review. Diagnostics (Basel) 14(24):2896. 10.3390/diagnostics14242896 39767257 10.3390/diagnostics14242896 PMC11675111

[14] Bisson T, Franz M, Kiehl TR et al (2025) A high-precision hierarchical registration approach for stain- and scanner-independent colocalization on whole slide images in histopathology. Health Inf Sci Syst 13:38. 10.1007/s13755-025-00353-7 40416515 10.1007/s13755-025-00353-7 PMC12102413

[15] Godau P, Kalinowski P, Christodoulou E et al (2025) Navigating prevalence shifts in image analysis algorithm deployment. Med Image Anal 102:103504. 10.1016/j.media.2025.103504 40020420 10.1016/j.media.2025.103504

[16] Gupta E, Gupta V (2025) Margin-aware optimized contrastive learning for enhanced self-supervised histopathological image classification. Health Inf Sci Syst 13:2. 10.1007/s13755-024-00316-4 39619405 10.1007/s13755-024-00316-4 PMC11607309

[17] Lingle W, Erickson BJ, Zuley ML et al (2016) The cancer genome atlas breast invasive carcinoma collection (TCGA-BRCA) (Version 3) [Data set]. Cancer Imaging Arch. 10.7937/K9/TCIA.2016.AB2NAZRP

[18] Lindgren CM, Adams DW, Kimball B et al (2021). Simplified and unified access to cancer proteogenomic data. 10.1021/acs.jproteome.0c00919 10.1021/acs.jproteome.0c00919 PMC8022323 33560848

[19] Thennavan A, Beca F, Xia Y et al (2021) Molecular analysis of TCGA breast cancer histologic types. Cell Genomics 1(3):100067. 10.1016/j.xgen.2021.100067 35465400 10.1016/j.xgen.2021.100067 PMC9028992

[20] Krug K, Jaehnig EJ, Satpathy S et al (2020) Proteogenomic landscape of breast cancer tumorigenesis and targeted therapy. Cell 183(5):1436–145631. 10.1016/j.cell.2020.10.036 33212010 10.1016/j.cell.2020.10.036 PMC8077737

[21] Brussee S, Valkema PA, Weijer JA, Doeleman T, Schrader AM, Kers J (2025) Pathbench-mil: A comprehensive automl and benchmarking framework for multiple instance learning in histopathology. arXiv preprint arXiv:2512.17517

[22] Garcia-Gutierrez J (2025) CLAiMem-ALL. https://github.com/BIGS-investigacion/CLAiMem-ALL.git

[23] Garcia-Gutierrez J (2025) CLAiMem-ALL. https://github.com/BIGS-Investigacion/PathBench-MIL.git

[24] Pedregosa F, Varoquaux G, Gramfort A et al (2011) Scikit-learn: machine learning in python. J Mach Learn Res 12:2825–2830

[25] Shao Z, Bian H, Chen Y et al (2021) Transmil: Transformer based correlated multiple instance learning for whole slide image classification. Adv Neural Inf Process Syst 34:2136–2147

[26] Li B, Li Y, Eliceiri KW (2021) Dual-stream multiple instance learning network for whole slide image classification with self-supervised contrastive learning. In: Proceedings of the IEEE/CVF conference on computer vision and pattern recognition, pp 14318–14328 10.1109/CVPR46437.2021.01409 PMC8765709 35047230

[27] Akiba T, Sano S, Yanase T, Ohta T, Koyama M (2019) Optuna: a next-generation hyperparameter optimization framework. In: The 25th ACM SIGKDD international conference on knowledge discovery & data mining, pp 2623–2631

[28] Macenko M, Niethammer M, Marron JS et al (2009) A method for normalizing histology slides for quantitative analysis. In: 2009 IEEE international symposium on biomedical imaging: from nano to macro. IEEE, pp 1107–1110. 10.1109/ISBI.2009.5193250

[29] Dolezal JM, Kochanny S, Dyer E et al (2024) Slideflow: deep learning for digital histopathology with real-time whole-slide visualization. BMC Bioinform 25(1):134. 10.1186/s12859-024-05758-x 10.1186/s12859-024-05758-x PMC10967068 38539070

[30] Ginter PS, Idress R, D’Alfonso TM, Fineberg S, Jaffer S, Sattar AK, Chagpar A, Wilson P, Harigopal M (2021) Histologic grading of breast carcinoma: a multi-institution study of interobserver variation using virtual microscopy. Mod Pathol 34(4):701–709. 10.1038/s41379-020-00698-2 33077923 10.1038/s41379-020-00698-2 PMC7987728

[31] Landis JR, Koch GG (1977) The measurement of observer agreement for categorical data. Biometrics 33(1):159–174 843571

[32] Shamai G, Schley R, Cretu A et al (2024) Clinical utility of receptor status prediction in breast cancer and misdiagnosis identification using deep learning on hematoxylin and eosin-stained slides. Commun Med 4(1):276. 10.1038/s43856-024-00695-5 39706861 10.1038/s43856-024-00695-5 PMC11661999

[33] Jang W, Lee J, Park K et al (2024) Molecular classification of breast cancer using weakly supervised learning. Cancer Res Treat 57(1):116–125. 10.4143/crt.2024.113 38938010 10.4143/crt.2024.113 PMC11729310

[34] Jahanifar M, Raza M, Xu K et al (2025) Domain generalization in computational pathology: Survey and guidelines. ACM Comput Surv 57(11). 10.1145/3724391

[35] Tellez D, Litjens G, Bándi P et al (2019) Quantifying the effects of data augmentation and stain color normalization in convolutional neural networks for computational pathology. Med Image Anal 58:101544. 10.1016/j.media.2019.101544 31466046 10.1016/j.media.2019.101544

[36] Hu EJ, Shen Y, Wallis P et al (2022) LoRA: Low-rank adaptation of large language models. In: International conference on learning representations

[37] Li Z, Ren K, Jiang X, Shen Y, Zhang H, Li D (2023) SIMPLE: Specialized model-sample matching for domain generalization. In: The eleventh international conference on learning representations

[38] Lee G, Jang W, Kim J et al. Domain generalization using large pretrained models with mixture-of-adapters. 10.1109/WACV61041.2025.00801

