# Supplementary material for: Domain generalisation challenges in breast cancer molecular classification using foundation models: a cross-cohort exploratory study
Source: Med Biol Eng Comput. 2026 May 11;64(6):2321–31. doi: 10.1007/s11517-026-03590-4 (PMC13269319; doi:10.1007/s11517-026-03590-4)
Supplement: Supplementary file 1 — (pdf 1.76 MB) [file 11517_2026_3590_MOESM1_ESM.pdf]

Supplementary Information

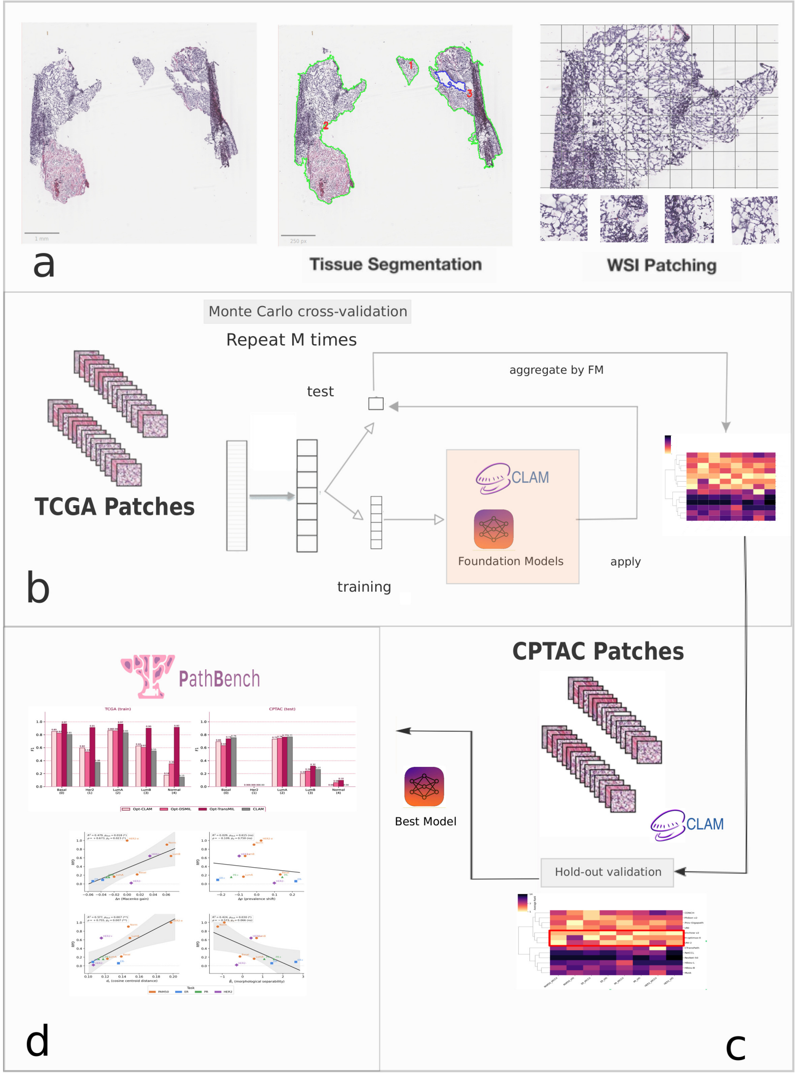

Fig. 1: Overview of the methodological pipeline: patch extraction (a), internal Monte Carlo cross-validation on TCGA (b), external hold-out validation on CPTAC with selection of the best FM-based model (c) and domain generalisation study (d).

Table 1: Foundation models evaluated in this study [11].

Name	Release	WSIs	Tiles	Architecture	Training Data
CTransPath [1]	Dec 2021	32K	16M	Swin-T	TCGA, PAIP
RetCCL [2]	Dec 2021	32K	16M	ResNet-50	TCGA, PAIP
CONCH [3]	Jul 2023	21K	16M	ViT-B	in-house
UNI [4]	Aug 2023	100K	100M	ViT-L	in-house
Prov-GigaPath [5]	May 2024	170K	1.4B	ViT	in-house
Hibou-B [6]	Jun 2024	1.1M	510M	ViT-B	in-house
Hibou-L [6]	Jun 2024	1.1M	1.2B	ViT-L	in-house
H-optimus-0 [7]	Jul 2024	500K	>100M	ViT-G	in-house
Virchow v2 [8]	Aug 2024	3.1M	2B	ViT-H	in-house
Phikon v2 [9]	Sep 2024	58.4K	456M	ViT-L	PANCAN-XL
Musk [10]	Jan 2025	33K	50M	BEiT3	TCGA
UNI-2 [4]	Jan 2025	350K	200M	ViT-H	in-house

Table 2: CLAM training parameters with early stopping (patience: 20 epochs, minimum delta: 0.001).

Parameter	MCCV	HO
seed	42	42
dropout	0.7	0.7
learning rate	0.0001	0.0001
weight decay	0.0001	0.0001
bag loss	cross-entropy	cross-entropy
instance loss	cross-entropy	cross-entropy
patches per bag	64	64
model size	big	big
training fraction	0.8	0.85
validation fraction	0.1	0.15
test fraction	0.1	0

Table 3: Best hyperparameter configurations found by Optuna for each task and MIL model in validation set.

**Task**	**Model**	z_{dim}	dropout
ER	CLAM	233	0.737
	DSMIL	57	0.747
	TransMIL	499	0.565
HER2	CLAM	317	0.756
	DSMIL	121	0.514
	TransMIL	325	0.534
PR	CLAM	33	0.543
	DSMIL	329	0.475
	TransMIL	439	0.652
PAM50	CLAM	255	0.549
	DSMIL	311	0.631
	TransMIL	472	0.597

Table 4: Performance per MIL aggregator. TCGA: hold-out test score (no stain normalisation); CPTAC: external test score (no stain normalisation). RPD: relative performance drop.

**MIL**	**Task**	**Class**	**Metric**	**TCGA**	**CPTAC**	RPD_c
CLAM	PAM50	Basal-like	F1	0.852	0.693	+0.187
		HER2-enriched		0.598	0.000	+1.000
		Luminal A		0.865	0.732	+0.154
		Luminal B		0.629	0.200	+0.682
		Normal-like		0.179	0.000	+1.000
	ER	ER-negative	PR-AUC	0.670	0.698	−0.042
		ER-positive		0.955	0.881	+0.077
	PR	PR-negative	PR-AUC	0.901	0.764	+0.152
		PR-positive		0.962	0.822	+0.145
	HER2	HER2-negative	PR-AUC	0.983	0.925	+0.059
		HER2-positive		0.728	0.176	+0.758
DSMIL	PAM50	Basal-like	F1	0.824	0.633	+0.232
		HER2-enriched		0.538	0.000	+1.000
		Luminal A		0.862	0.745	+0.136
		Luminal B		0.607	0.242	+0.601
		Normal-like		0.353	0.062	+0.824
	ER	ER-negative	PR-AUC	0.810	0.713	+0.120
		ER-positive		0.978	0.854	+0.127
	PR	PR-negative	PR-AUC	0.876	0.788	+0.101
		PR-positive		0.959	0.811	+0.154
	HER2	HER2-negative	PR-AUC	0.930	0.925	+0.005
		HER2-positive		0.329	0.123	+0.626
TransMIL	PAM50	Basal-like	F1	0.969	0.739	+0.237
		HER2-enriched		0.912	0.000	+1.000
		Luminal A		0.966	0.765	+0.208
		Luminal B		0.902	0.317	+0.649
		Normal-like		0.915	0.098	+0.893
	ER	ER-negative	PR-AUC	0.859	0.763	+0.112
		ER-positive		0.986	0.913	+0.074
	PR	PR-negative	PR-AUC	0.969	0.741	+0.235
		PR-positive		0.985	0.805	+0.183
	HER2	HER2-negative	PR-AUC	0.904	0.904	0.000
		HER2-positive		0.244	0.111	+0.545

Table 5: Effect of stain normalisation on per-class performance by MIL technique. Scores evaluated on CPTAC (external test set).

**MIL**	**Task**	**Class**	**None**	**Macenko**	Δn_c
CLAM	PAM50	Basal-like	0.693	0.682	−0.011
		HER2-enriched	0.000	0.000	+0.000
		Luminal A	0.732	0.740	+0.008
		Luminal B	0.200	0.386	+0.186
		Normal-like	0.000	0.103	+0.103
	ER	ER-negative	0.698	0.733	+0.035
		ER-positive	0.881	0.911	+0.030
	PR	PR-negative	0.764	0.733	−0.031
		PR-positive	0.822	0.758	−0.064
	HER2	HER2-negative	0.925	0.933	+0.008
		HER2-positive	0.176	0.234	+0.058
DSMIL	PAM50	Basal-like	0.633	0.694	+0.061
		HER2-enriched	0.000	0.000	+0.000
		Luminal A	0.745	0.732	−0.013
		Luminal B	0.242	0.246	+0.004
		Normal-like	0.062	0.160	+0.098
	ER	ER-negative	0.713	0.685	−0.028
		ER-positive	0.854	0.873	+0.019
	PR	PR-negative	0.788	0.771	−0.017
		PR-positive	0.811	0.828	+0.017
	HER2	HER2-negative	0.925	0.917	−0.008
		HER2-positive	0.123	0.140	+0.017
TransMIL	PAM50	Basal-like	0.739	0.735	−0.004
		HER2-enriched	0.000	0.000	+0.000
		Luminal A	0.765	0.710	−0.055
		Luminal B	0.317	0.329	+0.012
		Normal-like	0.098	0.080	−0.018
	ER	ER-negative	0.763	0.598	−0.165
		ER-positive	0.913	0.751	−0.162
	PR	PR-negative	0.741	0.705	−0.036
		PR-positive	0.805	0.757	−0.048
	HER2	HER2-negative	0.904	0.923	+0.019
		HER2-positive	0.111	0.141	+0.030

Table 6: Class prevalence in TCGA and CPTAC cohorts with calculated prevalence shifts.

**Task**	**Class**	$p_{\mathbf{TCGA}}$	$p_{\mathbf{CPTAC}}$	Δp_c
PAM50	Basal-like	0.170	0.302	+0.132
	HER2-enriched	0.076	0.101	+0.025
	Luminal A	0.525	0.450	−0.075
	Luminal B	0.179	0.114	−0.066
	Normal-like	0.049	0.034	−0.016
ER	ER-negative	0.160	0.380	+0.220
	ER-positive	0.840	0.620	−0.220
PR	PR-negative	0.312	0.455	+0.143
	PR-positive	0.688	0.545	−0.143
HER2	HER2-negative	0.793	0.891	+0.099
	HER2-positive	0.207	0.108	−0.099

Table 7: Cosine centroid distances per MIL model and mean (d_c) between TCGA and CPTAC class centroids in Virchow v2 feature space.

Task	Class	CLAM	DSMIL	TransMIL	d_c
PAM50	Basal-like	0.099	0.222	0.096	0.139
PAM50	HER2-enriched	0.118	0.365	0.109	0.197
PAM50	Luminal A	0.096	0.184	0.087	0.123
PAM50	Luminal B	0.123	0.204	0.121	0.149
PAM50	Normal-like	0.103	0.232	0.106	0.147
ER	ER-negative	0.096	0.208	0.102	0.136
ER	ER-positive	0.098	0.125	0.093	0.105
PR	PR-negative	0.093	0.133	0.111	0.112
PR	PR-positive	0.100	0.139	0.114	0.118
HER2	HER2-negative	0.091	0.145	0.082	0.106
HER2	HER2-positive	0.098	0.153	0.096	0.115

Table 8: Mean histomorphological feature values for TCGA and CPTAC patches ($n = 25$ patches per class per cohort). Values are means of the two pathologists' scores.

Task	Class	Cohort	Tubule Formation	Nuclear Pleomorphism	Mitotic Activity	Tumour Necrosis	Lymphocytic Infiltration	PMN Infiltration
PAM50	Basal-like	CPTAC	2.76	2.14	0.48	0.46	0.98	0.60
		TCGA	2.72	2.88	0.58	0.56	0.54	0.78
	HER2-enriched	CPTAC	2.76	1.96	0.60	0.48	0.76	0.52
		TCGA	2.82	2.78	0.38	0.48	0.96	0.72
	Luminal A	CPTAC	2.14	1.42	0.02	0.36	0.50	0.06
		TCGA	1.64	1.52	0.12	0.00	0.56	0.12
	Luminal B	CPTAC	2.38	1.76	0.26	0.12	0.88	0.32
		TCGA	2.20	2.26	0.28	0.10	0.80	0.22
	Normal-like	CPTAC	2.62	1.96	0.28	0.26	0.80	0.32
		TCGA	1.36	1.34	0.00	0.00	0.80	0.04
ER	ER-negative	CPTAC	2.80	2.66	0.68	0.44	0.86	0.54
		TCGA	2.72	2.60	0.30	0.54	0.62	0.76
	ER-positive	CPTAC	2.44	1.94	0.26	0.24	0.90	0.24
		TCGA	2.40	2.20	0.12	0.06	0.94	0.26
PR	PR-negative	CPTAC	2.68	2.10	0.44	0.44	0.84	0.56
		TCGA	2.72	2.50	0.36	0.72	0.66	0.78
	PR-positive	CPTAC	2.42	1.72	0.16	0.22	0.84	0.38
		TCGA	2.34	1.90	0.08	0.12	0.64	0.32
HER2	HER2-negative	CPTAC	2.58	1.86	0.30	0.26	0.94	0.48
		TCGA	2.50	2.36	0.24	0.16	0.48	0.34
	HER2-positive	CPTAC	2.58	2.22	0.38	0.34	0.82	0.46
		TCGA	2.62	2.78	0.28	0.36	0.56	0.44

Table 9: Inter-rater reliability between pathologists across $n = 275$ patches. Patches were aligned by image identifier prior to computing agreement. Weighted κ_w (linear weights) is reported for ordinal features; Cohen's κ for binary features. Interpretation follows Landis & Koch (1977) [12].

Feature	Statistic	n	Value
Ordinal features (weighted κ_w, linear weights)			
Tubule Formation	κ_w	275	0.289 (fair)
Nuclear Pleomorphism	κ_w	275	0.285 (fair)
Mitotic Activity	κ_w	275	0.152 (slight)
Binary features (Cohen's κ)			
Necrosis	κ	275	0.177 (slight)
Lymphocytic Infiltrate	κ	275	0.185 (slight)
PMN Infiltrate	κ	275	0.321 (fair)

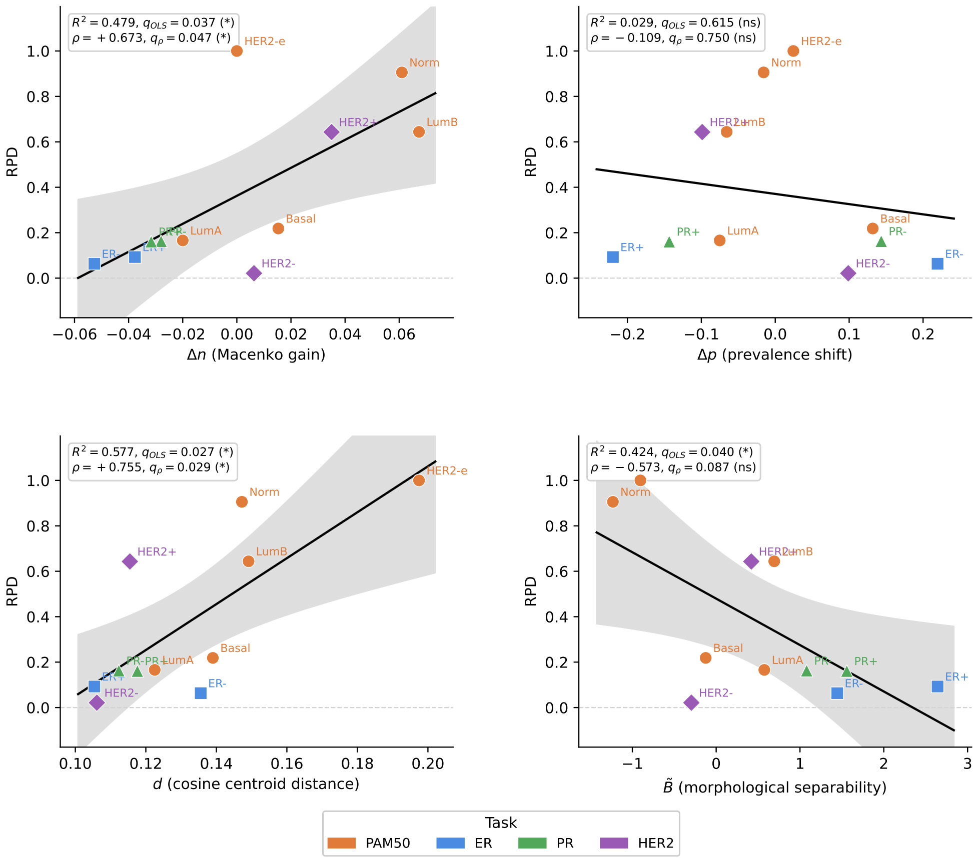

Fig. 2: Univariate regressions to explain RPD from domain shift factors. Each panel shows the relationship between RPD and a factor. Points are coloured by classification task (PAM50, ER, PR, HER2). The solid line is the OLS fit; the shaded region is the 95% confidence interval.

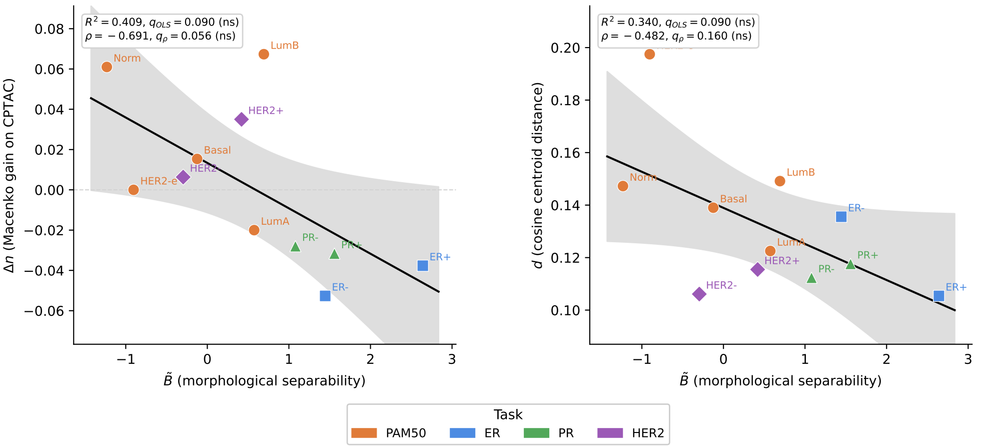

Fig. 3: Collinearity analysis between $\tilde{B}$ (morphological separability) and the two significant univariate factors (Δn on the left, d on the right). Points are coloured by classification task (PAM50, ER, PR, HER2). The solid line is the OLS fit; the shaded region is the 95% confidence interval.

References

[1] Wang, X., Yang, S., Zhang, J., *et al.*: Transformer-based unsupervised contrastive learning for histopathological image classification. Medical Image Analysis **81**, 102559 (2022) https://doi.org/10.1016/j.media.2022.102559

[2] Wang, X., Du, Y., Yang, S., *et al.*: RetCCL: Clustering-guided contrastive learning for whole-slide image retrieval. Medical Image Analysis **83**, 102645 (2023) https://doi.org/10.1016/j.media.2022.102645

[3] Lu, M.Y., Chen, B., Williamson, D.F.K., *et al.*: A visual-language foundation model for computational pathology. Nature Medicine **30**, 863–874 (2024) https://doi.org/10.1038/s41591-024-02856-4

[4] Chen, R.J., Ding, T., Lu, M.Y., et al.: Towards a general-purpose foundation model for computational pathology. Nature Medicine **30** (2024) https://doi.org/10.1038/s41591-024-02857-3

[5] Xu, H., Usuyama, N., Bagga, J., *et al.*: A whole-slide foundation model for digital pathology from real-world data. Nature (2024) https://doi.org/10.1038/s41586-024-07441-w

[6] Nechaev, D., Pchelnikov, A., Ivanova, E.: Hibou: A Family of Foundational Vision Transformers for Pathology (2024)

[7] Saillard, C., Jenatton, R., Llinares-López, F., Mariet, Z., Cahané, D., Durand, E., Vert, J.-P.: H-optimus-0 (2024). https://github.com/bioptimus/releases/tree/main/models/h-optimus/v0

[8] Zimmermann, E., Vorontsov, E., Viret, J., et al.: Virchow2: Scaling Self-Supervised Mixed Magnification Models in Pathology (2024)

[9] Filiot, A., Jacob, P., Kain, A.M., et al.: Phikon-v2, A large and public feature extractor for biomarker prediction (2024). https://arxiv.org/abs/2409.09173

[10] Xiang, J., Wang, X., Zhang, X., *et al.*: A vision–language foundation model for precision oncology. Nature (2025) https://doi.org/10.1038/s41586-024-08378-w

[11] Wölflein, G., Myles, C.: Pathology Feature Extractors and Foundation Models. https://github.com/georg-wolflein/pathology-foundation-models (2025)

[12] Landis, J.R., Koch, G.G.: The measurement of observer agreement for categorical data. Biometrics **33**(1), 159–174 (1977)
